# Initial Experience on Hyperpolarized [1-^13^C]Pyruvate MRI Multicenter Reproducibility—Are Multicenter Trials Feasible?

**DOI:** 10.3390/tomography8020048

**Published:** 2022-03-01

**Authors:** Nikolaj Bøgh, Jeremy W. Gordon, Esben S. S. Hansen, Robert A. Bok, Jakob U. Blicher, Jasmine Y. Hu, Peder E. Z. Larson, Daniel B. Vigneron, Christoffer Laustsen

**Affiliations:** 1 The MR Research Center, Department of Clinical Medicine, Aarhus University, 8200 Aarhus, Denmark; esben@clin.au.dk (E.S.S.H.); cl@clin.au.dk (C.L.); 2 Department of Radiology and Biomedical Imaging, University of California San Francisco, San Francisco, CA 94158, USA; jeremy.gordon@ucsf.edu (J.W.G.); robert.bok@ucsf.edu (R.A.B.); jasmine.hu@ucsf.edu (J.Y.H.); peder.larson@ucsf.edu (P.E.Z.L.); dan.vigneron@ucsf.edu (D.B.V.); 3 Center of Functionally Integrative Neuroscience, Aarhus University, 8000 Aarhus, Denmark; jbli@cfin.au.dk; 4 UC Berkeley—UCSF Graduate Program in Bioengineering, University of California San Francisco and University of California, Berkeley, CA 94720, USA

**Keywords:** magnetic resonance imaging, metabolism, hyperpolarized, pyruvate, brain, repeatability, multisite

## Abstract

Background: Magnetic resonance imaging (MRI) with hyperpolarized [1-^13^C]pyruvate allows real-time and pathway specific clinical detection of otherwise unimageable in vivo metabolism. However, the comparability between sites and protocols is unknown. Here, we provide initial experiences on the agreement of hyperpolarized MRI between sites and protocols by repeated imaging of same healthy volunteers in Europe and the US. Methods: Three healthy volunteers traveled for repeated multicenter brain MRI exams with hyperpolarized [1-^13^C]pyruvate within one year. First, multisite agreement was assessed with the same echo-planar imaging protocol at both sites. Then, this was compared to a variable resolution echo-planar imaging protocol. In total, 12 examinations were performed. Common metrics of ^13^C-pyruvate to ^13^C-lactate conversion were calculated, including the *k*_PL_, a model-based kinetic rate constant, and its model-free equivalents. Repeatability was evaluated with intraclass correlation coefficients (ICC) for absolute agreement computed using two-way random effects models. Results: The mean *k*_PL_ across all examinations in the multisite comparison was 0.024 ± 0.0016 s^−1^. The ICC of the *k*_PL_ was 0.83 (*p* = 0.14) between sites and 0.7 (*p* = 0.09) between examinations of the same volunteer at any of the two sites. For the model-free metrics, the lactate Z-score had similar site-to-site ICC, while it was considerably lower for the lactate-to-pyruvate ratio. Conclusions: Estimation of metabolic conversion from hyperpolarized [1-^13^C]pyruvate to lactate using model-based metrics such as *k*_PL_ suggests close agreement between sites and examinations in volunteers. Our initial results support harmonization of protocols, support multicenter studies, and inform their design.

## 1. Introduction

Hyperpolarization is an emerging research technology that can add dynamic metabolic imaging to the MRI toolbox by enhancing the signal of ^13^C-enriched molecules [1]. The hyperpolarized molecule and its metabolites are detected after intravenous administration. The observable metabolites of [1-^13^C]pyruvate are lactate, bicarbonate, and alanine, providing information on key metabolic pathways within a single scan. As increased glucose uptake and lactate production in the presence of sufficient oxygen, known as the Warburg effect, is often observed in cancer, MRI with hyperpolarized [1-^13^C]pyruvate can aid prediction of aggression and therapy response [2,3]. Accordingly, in addition to studies in healthy individuals [4,5], cancer was the focus of the initial neuroimaging studies in disease [6,7,8,9]. Preclinically, the technology has shown promise in cancer as well as non-cancerous conditions, including vascular, traumatic, and inflammatory disease [10,11,12,13].

The conversion of hyperpolarized [1-^13^C]pyruvate to lactate is quantifiable through mode-free approaches or kinetic fitting of the dynamic pyruvate and lactate images. Multisite trials or pooling of data should accelerate larger studies that will drive the technology to clinical use. However, it is unknown whether it suffers from the same reproducibility difficulties as some quantitative MRI technologies [14], or whether it is as reproducible as others such as diffusion weighted imaging [15]. Since the first trial in 2013 [16], over 600 human examinations have been performed. The studies have been single-site efforts of several different organs and pathologies with just a few patients for each [6,7,8,9], and differences in acquisition and quantification strategies make it difficult to aggregate data. Therefore, a recent whitepaper deemed multisite trials a prerequisite to successful translation of hyperpolarized MRI [17].

Good repeatability is warranted for collaborative trials; however, no data exist to evaluate the multisite comparability of MRI with hyperpolarized [1-^13^C]pyruvate. In order to prepare for the necessary large-scale multicenter studies, we conducted a traveling healthy volunteer pilot study to investigate the reproducibility of hyperpolarized brain imaging across sites using the same volunteers at both sites.

## 2. Materials and Methods

### 2.1. Study Design and Volunteers

Although coronavirus pandemic restrictions implemented soon after the initiation of this project in 2020 limited the travel and therefore the number of subjects in this multisite study, three healthy male volunteers (mean age of 34.7 years, span 29 to 38) were successfully imaged with hyperpolarized [1-^13^C]pyruvate MRI of the brain on 3T systems (MR750, GE Healthcare) at the two sites. They were asked to limit caffeine to two cups of coffee and avoid alcohol from the night before the examinations. The examinations at site 1 were performed from November 2019 to December 2019, while the examinations at site 2 were performed in November 2020. In total, 12 examinations were performed (3, 4, and 5 in each participant, respectively), of which one examination from each participant were published previously [12]. The studies were approved by the Institutional Review Board at the University of California, San Francisco, the Committee on Health Research Ethics for Central Denmark, and the Danish Medicines Agency.

### 2.2. Coil Comparison

Three different ^13^C coils were used in this study (please refer to the section below). To compare the similarity of hardware between sites, the signal-to-noise ratio (SNR) and B_1_^+^ profile of the coils employed were estimated on thermal phantoms. A spherical (Ø = 16 cm) gadolinium-doped dimethyl silicone phantom with natural ^13^C-abundance was used (GE Healthcare). Power calibration was performed using an FID-CSI sequence with a 90° hard pulse. The phantom experiments utilized spectral-spatial excitation of a single resonance with an echo-planar imaging (EPI) readout. The B_1_^+^ mapping was performed using a double-angle protocol (30°/60°) with a 5 cm slice thickness, 24 × 24 cm field of view (FOV), 16 × 16 matrix size, 3 s TR, and 20 min total scan time. The SNR experiments were performed with 10 slices of 2 cm thickness, 24 × 24 cm FOV, 16 × 16 matrix size, 62° flip angle, 0.75 s TR, and NEX = 400, yielding a total scan time of 5 min. The SNR was calculated as the mean signal across the phantom divided by signal standard deviation (SD) in a slice of noise located outside the phantom.

### 2.3. Hyperpolarized MRI

Pyruvate (1.47 g of [1-^13^C]pyruvic acid, Sigma-Aldrich) was hyperpolarized with 15 mM AH111501 (Syncom or GE Healthcare) in a 5T SPINlab (GE Healthcare) polarizer [1]. The sample was dissoluted with water, filtered, buffered, and pH, temperature, polarization, volume, and concentrations of pyruvate and radical were measured before injection (0.43 mL/kg chased by 20 mL of saline at 5 mL/s). Imaging was started 2 s after the end of saline flush, corresponding to before the arrival of the pyruvate bolus.

The multi-site examinations followed a single protocol with metabolite-selective spectral-spatial excitation (Protocol 1, Table 1). This protocol used a published EPI sequence [18] and comparable volume transceiver coils (home-built, UCSF-MGH at site 1; PulseTeq, UK at site 2). To evaluate agreement between differing protocols, another four examinations were performed using a variable-resolution EPI with different flip angles (Protocol 2, Table 1) using a 24-channel ^13^C receive coil (RAPID Biomedical) at site 1 [19]. The [1-^13^C]pyruvate center frequency was estimated from the proton frequency [20] and confirmed with a non-localized spectrum acquired after imaging. Before each imaging session, transmit power was calibrated on an ethylene glycol head phantom loading equally to a human head. Reference anatomical images were acquired using a spoiled gradient echo sequence (TR/TE = 7/2.4 ms, flip angle = 12°, matrix = 256 × 256, FOV = 256 × 256 mm^2^, 1.5 mm slices) just before ^13^C-imaging without moving the participant. Similarly, a B_0_ field map was obtained using an Iterative Decomposition of water and fat with Echo Asymmetry and Least squares estimation sequence (IDEAL IQ, 10.9 ms TR, 4.1 ms TE, 3 echo train length). Blood flow data were obtained using pseudo-continuous arterial spin labeling (3D, 3.6 mm^3^ isotropic resolution, 2025 ms post label-delay).

### 2.4. Data Reconstruction and Analysis

All ^13^C-data were reconstructed in MATLAB (MathWorks). The EPI data were phase corrected and Fourier transformed [18,19]. Multichannel data were coil combined with weights determined from the pyruvate data [22].

Model-based and model-free analyses were performed for quantification. Rate constants of pyruvate-to-lactate conversion (*k*_PL_) were fitted in a voxel-wise fashion using a two-site metabolic exchange model [23]. No assumptions were made regarding the input function, and only the forward reaction was considered. The initial *k*_PL_ estimate was 0.02 s^−1^. Fitting was performed using the Hyperpolarized MRI Toolbox (available at github.com/LarsonLab/hyperpolarized-mri-toolbox, doi: 10.5281/zenodo.1198915). We calculated the lactate-to-pyruvate ratio and the Z-score of lactate SDs from the whole-brain mean based on manually drawn regions of interest, representing frequently used model-free quantifications [5,9]. Region of interest (ROI) analyses were performed on ^13^C-images overlaid on anatomical reference scans in Horos (The Horos Project, horosproject.org) for the whole brain, the cortex, and deep white matter at the level of the ventricular bodies. The whole brain region excluded the sinuses and scalp but included the ventricles that are not expected to give raise to metabolic signal. SNR was calculated as whole brain signal divided by the SD of signal in a large region of noise outside the head.

In a sub-analysis, we adjusted for the echo time differences between the sequences of Protocol 1 and 2. This was based on a global T_2_* of pyruvate and lactate that was determined from the full width at half maximum in the non-localized, post-imaging spectra. Then, correction factors were determined using $S_{0}=\frac{S}{\exp\left(-\frac{TE}{T2*}\right)}\;$, where *S* is the observed signal and *S*_0_ is the corrected signal. In one case, a spectrum was not acquired, and the average correction factor for examinations under the same protocol was applied. The correction was not applied for the Z-score, as only the ^13^C-lactate signal is used.

### 2.5. Statistics

Data are presented as mean ± SD. Repeatability was assessed between sites, where all examinations made at a single site were averaged. In addition, repeatability was assessed between examinations of the same participant, regardless of the site at which the examination was performed. The intraclass correlation coefficient (ICC) for absolute agreement of the average of the cortical and deep white matter regions of interest was determined with a two-way random effects model as suggested by Koo et al. [24]. Generally, an ICC below 0.5 is considered poor, between 0.5 and 0.75 is considered moderate, and between 0.75 and 0.9 is considered good [24]. T-tests were used for comparison of means of SNR and apparent rate constants. Statistical analysis was performed in R (R Core Team 2014, R-project.org).

## 3. Results

### 3.1. Comparability of Coils

**Figure 1:**
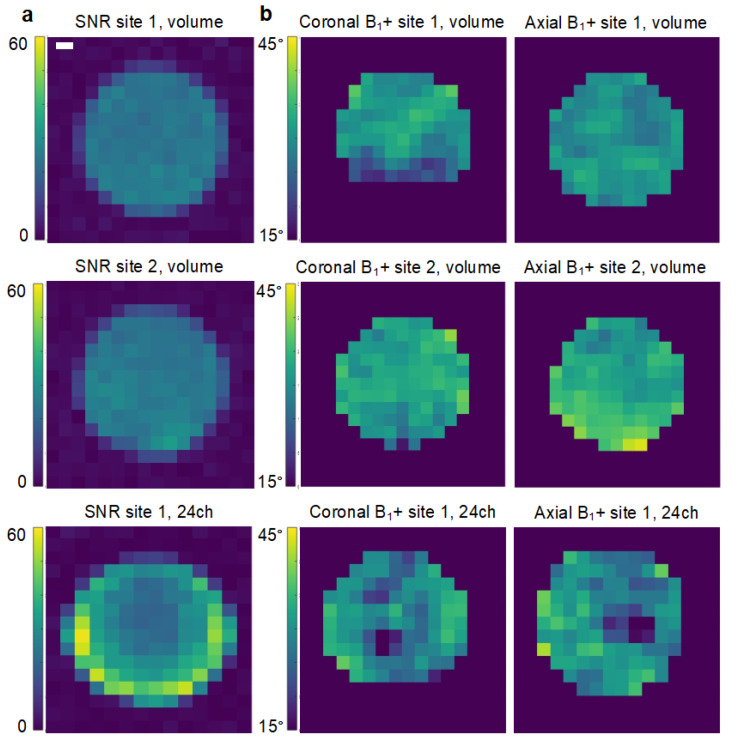
Comparison of the coils used at the two sites. The tests were performed on a natural abundance dimethyl silicone phantom by excitation of a single resonance with an echo-planar imaging readout. The signal-to-noise ratio (SNR, (**a**)) was determined over a 5 min scan. The B_1_^+^ profile (**b**) was determined using the double angle method. The white scale is 2 cm.

The phantom SNR was 21.7 ± 4.3 at site 1 and 22.2 ± 4.7 at site 2 using the volume transceiver coils. The SNR of the 24-channel coil used at site 1 was 33 ± 11. The B_1_^+^-fields of the volume transceivers were largely homogenous, while it was slightly more variable for the 24-channel setup (31 ± 2° vs. 33 ± 3° vs. 29 ± 5.7°). Maps of SNR and B_1_^+^ are presented in Figure 1.

### 3.2. Metadata of Human Examinations

**Figure 2:**
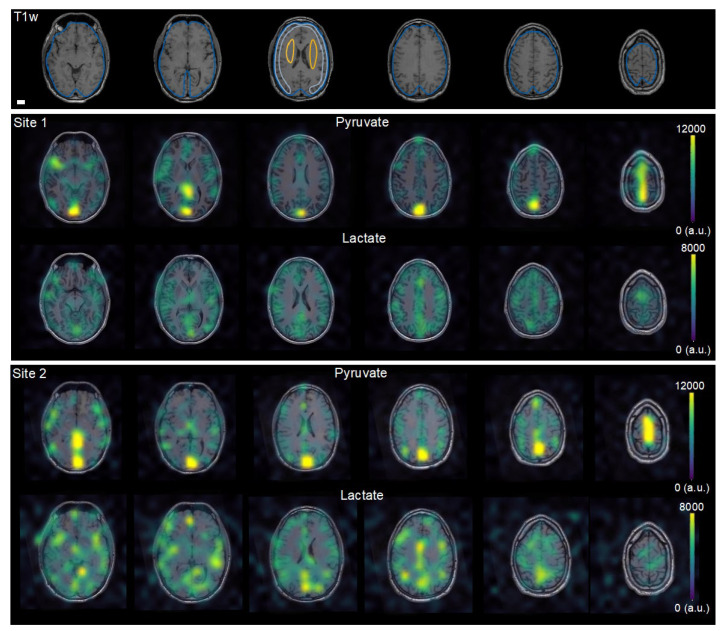
Comparison of hyperpolarized [1-^13^C]pyruvate MRI data from each site in the same participant. After injection of hyperpolarized [1-^13^C]pyruvate, dynamic images of [1-^13^C]pyruvate and [1-^13^C]lactate and were acquired in the same subjects at two sites. The six most cranial summed images are shown overlaid on T1 weighted (T1w) images from site 2, highlighting comparability between sites. Example regions of interest are shown for the whole brain (blue), deep white matter (yellow), and cortex (grey). The white scale equals 2 cm.

Time from dissolution to injection was 51.8 ± 1.9 s at site 1 versus 86.2 ± 23.3 s at site 2 (*N* = 12). In all examinations, the center frequency was prescribed within 20 Hz accuracy, well within the excitation passband. The mean B_0_ SD at the proton frequency over the entire brain was 23.7 ± 1.8 Hz under Protocol 1 (*n* = 6) and 40.4 ± 6.9 Hz under Protocol 2 (*n* = 4). As bicarbonate was only detected at site 1 using Protocol 2, which employed a more sensitive 24-channel ^13^C-coil, it was not considered further (the bicarbonate SNR was 2.4 ± 0.9 under Protocol 1). The in vivo SNR of pyruvate and lactate summed over time were 10.9 ± 4 and 13.8 ± 12.4 under Protocol 1 (*p* > 0.1 for the effect of site; *n* = 8). The brain-wide cerebral blood flow was 38.9 ± 7.4 mL/100 mL/min (*p* > 0.1 for site; *n* = 10). Data examples are presented in Figure 2, showing the large pyruvate pool within the vasculature, primarily the venous system, and lactate production predominantly confined to the metabolically active cortex as also observed in previous studies [4,5,18].

### 3.3. Multisite Agreement of Hyperpolarized MRI

**Figure 3:**
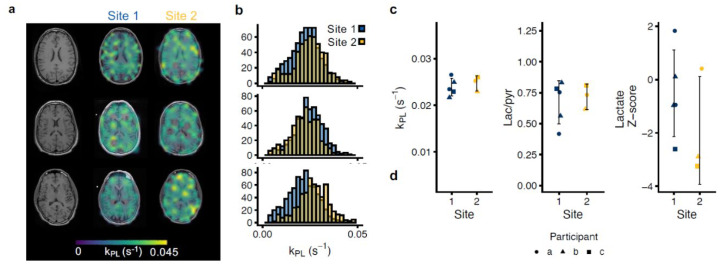
Multisite repeatability of hyperpolarized [1-^13^C]pyruvate MRI. Imaging was performed at two sites under the same protocol (two individuals scanned three times and one scanned twice). Examples of the apparent rate constant of pyruvate-to-lactate conversion ((**a**), *k*_PL_) are shown with corresponding histograms (**b**). Good site-to-site agreement was observed, particularly of the *k*_PL_ (**c**). The data are shown as individual observations of a whole brain region of interest average with one SD.

Our initial analyses focused on data acquired with the same protocol at two sites (Protocol 1, three scans in two participants and two in one participant; Figure 3, and Table 2). We observed no significant differences in lactate-to-pyruvate conversion metrics between the sites in any of the three regions of interest. The average *k*_PL_ across the whole brain, cortex, and deep white matter regions of interest at site 1 were 0.024 ± 0.0018, 0.025 ± 0.0019 and, 0.029 ± 0.0033 s^−1^, respectively, versus 0.024 ± 0.0021, 0.026 ± 0.0013, and 0.028 ± 0.0016 s^−1^, respectively, at site 2 (*p* > 0.2 for all comparisons). The average SD of *k*_PL_ within the whole brain ROI (538 ± 49 voxels at site 1, 463 ± 47 voxels at site 2) was 0.008 ± 0.0004 s^−1^ at site 1 versus 0.0085 ± 0.0003 s^−1^ at site 2 (*p* = 0.09). Within the cortex ROI, the average *k*_PL_ SD was 0.0069 ± 0.0009 s^−1^ at site 1 versus 0.0079 ± 0.0013 s^−1^ at site 2 (*p* = 0.4; 55 ± 5 versus 58 ± 3 voxels). Lastly, in the deep white matter ROI, it was 0.0053 ± 0.0006 s^−1^ at site 1 versus 0.006 ± 0.0005 s^−1^ at site 2 (*p* = 0.13; 32 ± 2 versus 29 ± 2 voxels). The site-to-site ICCs were 0.83 (*p* = 0.14), 0.64 (*p* = 0.27), and 0.76 (*p* = 0.2) for *k*_PL_, the lactate-to-pyruvate ratio, and the lactate Z-score, respectively. Under Protocol 1, the examination-to-examination ICCs, disregarding the site in the calculation, were 0.70 (*p* = 0.09) for *k*_PL_, 0.57 (*p* = 0.18) for the lactate-to-pyruvate ratio, and 0.59 (*p* = 0.04) for the lactate Z-score.

### 3.4. Inter-Protocol Variation of Hyperpolarized [1-^13^C]pyruvate MRI

**Figure 4:**
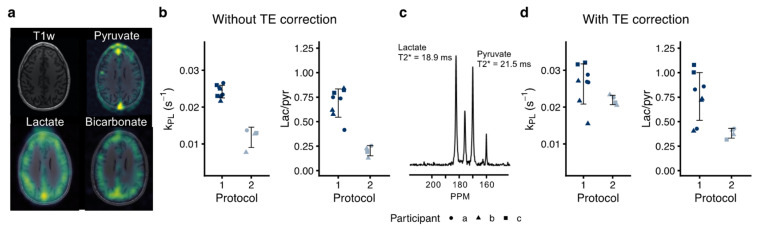
Agreement of pyruvate-to-lactate conversion with hyperpolarized [1-^13^C]pyruvate between two protocols. One individual was imaged five times, one four times, and one three times (12 injections in total, 9 at site 1, 3 at site 2). In addition to the single-resolution echo-planar imaging protocol presented above, the volunteers were imaged with a variable-resolution equivalent ((**a**), see Table 1). The whole-brain apparent rate constant (*k*_PL_) and the model-free whole-brain lac/pyr ratio show good repeatability within each protocol (**b**). However, the echo time difference between the protocols caused lower pyruvate-to-lactate conversion estimates using the variable-resolution protocol (Protocol 2). Independent T_2_* values of ^13^C-pyruvate and ^13^C-lactate were estimated from non-localized spectra acquired after imaging (example in (**c**)). These were used to adjust for the echo time differences between protocols. The correction improved the agreement of *k*_PL_ in particular but introduced more variation (**d**). The data are shown as individual observations, and the error bars represent one SD.

We investigated the repeatability of pyruvate-to-lactate conversion metrics between two protocols (Figure 4; *N* = 12). Initially, we found that Protocol 1 and 2 differed by about 40–50% in their estimation of pyruvate-to-lactate conversion. Protocol 2 used a multi-element coil and a variable-resolution EPI with longer echo time. To account for this, we performed a subsequent correction of T_2_* decay. The global T_2_* of pyruvate, measured from the non-localized spectra, was longer than that of lactate (15.2 ± 5.9 ms vs. 13.9 ± 5.1 ms). The T_2_* values were shorter with the multi-channel coil than with the volume transceivers (8.9 ± 1.6 ms vs. 18 ± 4.6 ms for pyruvate, *p* < 0.001; 7.5 ± 1.3 ms vs. 16.7 ± 3.4 ms for lactate, *p* < 0.001). This adjustment largely eliminated the difference observed for Protocol 2.

## 4. Discussion

The main finding of this study is the good agreement between sites in the same subjects demonstrating the feasibility of multi-institutional HP ^13^C-pyruvate MRI studies. The results also support multicenter collaborations combining the data from subjects being scanned at a single site and suggest potential for absolute quantification. These findings are similar to previous results of good same-day repeatability within a single site [25,26], as we found close agreement between examinations performed over a year, suggesting that the technology is robust to physiological or technical variations over this longer time period. Further, the observed 20–30% relative SDs of *k*_PL_ across the ROIs were similar between individuals and sites, and it was within the range of variation observed with [^18^F]2-fluoro-2-deoxy-D-glucose–positron emission tomography (^18^FDG-PET) in rats and phantoms [27,28]. Of interest, the change in some pathologies such as stroke and inflammation is expected to be significantly more than the relative variation observed here [11,12], while that may not always be the case in other diseases such as cancer [6,8,9], even though aggressive disease may have a distinct metabolic signature [9].

Adding valuable metabolic information to MRI, hyperpolarization is currently in clinical translation [17] and multiple single-site clinical trials are ongoing. Particularly useful in oncology, this technology allows quantification of cancerous metabolism and its response to therapy [17]. To facilitate translation, and to be truly useful, MRI with hyperpolarized [1-^13^C]pyruvate must be repeatable and comparable across sites. Here, we provide the initial multisite experiences, suggesting good agreement between two sites. However, when comparing different protocols, corrections may be needed, as further underlined by the lower apparent *k*_PL_ observed in a previous study in healthy volunteers [4]. In this study, echo time correction was needed to avoid confounding of the metabolic metrics by the different T_2_* of lactate and pyruvate [29,30]. The correction allowed comparison of single and variable resolution data obtained with different readouts and echo times. However, the T_2_*-estimation from non-localized spectra introduced additional variation from field inhomogeneities, including from outside the brain, as it only provided global estimates. For future studies, local T_2_* values may be estimated with multi-echo or chemical shift ^13^C-imaging [30] or extrapolated from the proton B_0_ maps. This would allow more accurate estimation of T_2_* and local corrections. In extension, shim quality should ideally be comparable between protocols, hardware, and sites, although our data show that minor differences can be retrospectively accounted for. Of note, the finer resolution data have less partial volume effects, which may still affect the reported repeatability measures in ways that we cannot correct for.

It follows that protocol harmonization and phantom validation of the experimental setup (signal-to-noise ratio, B_1_ calibration, and B_0_) are important in collaborative efforts. For example, imprecise power calibration would lead to errors in *k*_PL_ estimates. This calls for standardized experiments that can be used to assess and correct the biases between protocols and setups. It is particularly important to point out that a harmonized hyperpolarized phantom for qualifying equipment and protocols would ease this process. The EPI sequence used in this study [18] has been applied widely in multiple human studies and as such represents an appealing option for future multicenter clinical trials. The imaging readout can be adjusted in the pre-scan using proton data; however, the EPI suffers from long echo times and so spiral readouts could prove useful in this regard. Further, it is worth noting that it is not fully understood what the optimal timing or flip angle schemes should be, or how point-spread bleeding of signals between voxels influences our metrics, and thus future studies need to evaluate this in better detail.

The lactate-to-pyruvate ratio, the lactate Z-score, and the *k*_PL_ are popular metrics that attempt to quantify pyruvate-to-lactate conversion. In particular, the lactate-to-pyruvate ratio is an appealing model-free metric [31]. Although these two metrics are proportional, the *k*_PL_-fitting accounts for the differences in flip angle, whereas the lactate-to-pyruvate ratio is usually not corrected for the applied flip angles. This probably makes *k*_PL_ better suited for data aggregation across protocols. The Z-score had similar repeatability to *k*_PL_ in our analysis. As only one metabolite is analyzed, the Z-score has the advantage of being robust to differences in flip angles and T_2_* decay between resonances. However, there seemed to be sizeable variation between volunteers, and, important in a comparability context, the Z-score is sensitive to the coil profile. The straightforward Z-score performs best with homogenous coils; alternatively, coil profile correction is warranted [32], which our data did not allow as sensitivity profiles were not acquired. Using a strategy suggested for parametric mapping, an individual Z-score of the *k*_PL_ may provide a viable compromise that is less sensitive to echo time differences, flip angles, and coil sensitivity [14].

We observed good agreement across examinations performed at different institutions; however, this feasibility study was limited by its small size. Another potential limitation is the fact that the participants were all healthy, young men and thus care must be taken when extending our findings to more diverse populations with varying ethnicity, gender, age, and health status. This is especially a concern in elderly patients, which are likely to be the main users of hyperpolarized MRI. However, it is important to point out that the current study design is not practical for patients or the elderly, and thus multisite repeatability studies are often performed in healthy, young individuals. Due to its small size and uncertain statistical estimates, the current study is best utilized as a primer for future multicenter studies. For example, if an ICC of 0.8 of the *k*_PL_ between sites is to be confirmed with ± 0.2 precision and a 95% confidence level, 14 volunteers should be imaged repeatedly at two sites, or 10 volunteers at three sites [33].

Repeatability between vendors may be a concern in quantitative MRI. We were unable to compare data acquired on systems from more than a single vendor in this pilot study. Although the equipment used in this study is the currently most prevalent in hyperpolarized ^13^C MRI, it is important to note that including other vendor systems in the coming multicenter studies would greatly improve their impact. It is worth mentioning that we used coils from different vendors, which did not appear to cause variation. Lastly, we provide no estimates on the repeatability of pyruvate-to-bicarbonate conversion, but we expect that it is comparable to the repeatability of pyruvate-to-lactate conversion.

## 5. Conclusions

Collaborative multisite efforts are a critical step towards overcoming translational barriers and advancing hyperpolarized MRI to benefit patients. By suggesting good repeatability and shedding light on the potential pitfalls, the data provided here clear the path for further multisite studies with hyperpolarized MRI.

## Figures and Tables

**Table 1:** Overview of the different protocols employed.

Protocol	Site	Excitation	Flip-Angles, Pyruvate/ Metabolites	Read Out	TR/TE (ms)	Voxel Size ^†^, Pyruvate/ Metabolites (mm^3^)	Temporal Resolution (s)	Coil	*N*
1	1 + 2	Spectral-spatial	10°/40° *	EPI	62.5/ 21.7	15 × 15 × 15/ 15 × 15 × 15	3	Birdcage volume	8
2	1	Spectral-spatial	20°/30°	EPI	125/ 30.7	7.5 × 7.5 × 15/ 15 × 15 × 15	3	Birdcage transmit + 24-ch receive	4

* In a single case, a 20°/30° flip angle scheme was used. ^†^ The effective resolution in the phase-encode dimensions is 1.04 coarser due to T_2_* decay [21].

**Table 2:** Average metabolic metrics in regions of interest compared between the two sites (*n* = 5 at site 1, *n* = 3 at site 2) using the same single-resolution protocol (Protocol 1).

	Protocol 1					
	Site 1			Site 2		
	*k*_PL_	Lactate/ Pyruvate	Lactate Z-Score	*k*_PL_	Lactate/ Pyruvate	Lactate Z-Score
Whole brain	0.024 ± 0.0018	0.67 ± 0.17	-	0.024 ± 0.0021	0.63 ± 0.2	-
Cortex	0.025 ± 0.0019	0.75 ± 0.16	−0.5 ± 1.63	0.026 ± 0.0013	0.73 ± 0.32	−1.66 ± 1.72
Deep white matter	0.029 ± 0.0033	0.96 ± 0.22	1.56 ± 1.5	0.028 ± 0.0016	0.96 ± 0.23	0.95 ± 1.4

## Data Availability

The data presented in this study are available on request from the corresponding author. The data are not publicly available due to GDPR regulations.
